# The Achilles’ heel of cancer survivors: fundamentals of accelerated cellular senescence

**DOI:** 10.1172/JCI158452

**Published:** 2022-07-01

**Authors:** Shameel Shafqat, Evelyn Arana Chicas, Areez Shafqat, Shahrukh K. Hashmi

**Affiliations:** 1 Medical College, Aga Khan University, Karachi, Pakistan.; 2 Department of Surgery, University of Rochester Medical Center, Rochester, New York, USA.; 3 College of Medicine, Alfaisal University, Riyadh, Saudi Arabia.; 4 Department of Internal Medicine, Mayo Clinic, Rochester, Minnesota, USA.; 5 Clinical Affairs, Khalifa University, Abu Dhabi, United Arab Emirates.; 6 Department of Medicine, Sheikh Shakhbout Medical City, Abu Dhabi, United Arab Emirates.

## Abstract

Recent improvements in cancer treatment have increased the lifespan of pediatric and adult cancer survivors. However, cancer treatments accelerate aging in survivors, which manifests clinically as the premature onset of chronic diseases, such as endocrinopathies, osteoporosis, cardiac dysfunction, subsequent cancers, and geriatric syndromes of frailty, among others. Therefore, cancer treatment–induced early aging accounts for significant morbidity, mortality, and health expenditures among cancer survivors. One major mechanism driving this accelerated aging is cellular senescence; cancer treatments induce cellular senescence in tumor cells and in normal, nontumor tissue, thereby helping mediate the onset of several chronic diseases. Studies on clinical monitoring and therapeutic targeting of cellular senescence have made considerable progress in recent years. Large-scale clinical trials are currently evaluating senotherapeutic drugs, which inhibit or eliminate senescent cells to ameliorate cancer treatment–related aging. In this article, we survey the recent literature on phenotypes and mechanisms of aging in cancer survivors and provide an up-to-date review of the major preclinical and translational evidence on cellular senescence as a mechanism of accelerated aging in cancer survivors, as well as insight into the potential of senotherapeutic drugs. However, only with time will the clinical effect of senotherapies on cancer survivors be visible.

Cancer survival times have increased annually owing to advances in early detection and treatment that prolong patient survival. However, increasing survivorship has underscored the observation that cancer survivors develop age-related diseases prematurely, which cause significant morbidity, health expenditures, and mortality. Many cancer survivors have been exposed to chemotherapy, radiotherapy, or both; despite eradicating cancer cells, these therapies also damage normal cells to accelerate biologic aging, such that a discrepancy exists between their biologic and chronologic age (1). Considerable data exist regarding the phenotypes of accelerated aging. However, mechanical and molecular uncertainties have limited the study of these manifestations in a clinical context. Our Review discusses accelerated aging phenotypes in cancer survivors and the cellular mechanisms underpinning these phenomena. We then discuss the translational evidence on how accelerated aging phenotypes, mainly related to senescence, are being targeted while highlighting areas of uncertainty for future research to address.

## Accelerated phenotypic aging in cancer survivors

Aging is a normal process of life characterized by progressive loss of fitness that renders individuals more vulnerable to diseases and treatment complications, medical or surgical. Aging results from incremental accumulations in cellular and molecular damage manifested phenotypically as functional decline and a reduced ability to maintain tissue homeostasis in response to stressors, i.e., frailty. Such stressors certainly include cancer and cancer treatment; accelerated aging phenotypes are detected in childhood and adult cancer survivors (Table 1).

Childhood cancer survivors (CCS) develop age-related diseases faster than their healthy counterparts (2, 3). For instance, CCS are significantly more likely than siblings to develop a chronic condition (relative risk [RR], 3.3) or a life-threatening condition (RR, 8.2) (4). The cumulative incidence of a chronic health condition among survivors is 73.4% (4), and the prevalence of second malignant neoplasms is nearly 8% (5). Lastly, survivors’ estimated life expectancy is 30% less than that of the general population (6). The St. Jude Lifetime Cohort study (SJLIFE) used the Fried criteria for frailty (7) and showed frailty in 31% of female and 12.9% of male CCS, whereas no age-matched controls without a cancer history were frail. Moreover, in comparison with non-frail individuals, frailty increased mortality risk (hazard ratio [HR], 2.26) and chronic disease onset (RR, 2.26) (8). The characteristically high frailty rate in CCS may be due to the rigorous treatment regimens they are exposed to (9, 10). Indeed, dose intensification is expected for childhood cancers, partly because of their genetic complexity and the ability of a child’s bone marrow (BM) to recover (9).

In contrast, clinical research on accelerated aging in elderly cancer survivors is limited; only 5% of NIH-funded survivorship studies investigate these phenomena in older adults (11). Therefore, older cancer survivors are severely underrepresented in cancer research, and more investigations into the effects of cancer treatment in this population are warranted. A step forward in this regard was a study conducted by Siddique et al., which showed increased frailty in adult cancer survivors compared with those without a cancer history (12). Lintermans et al. assessed grip strength in women receiving aromatase inhibitors 6 months and 12 months after initiation of therapy and showed a significant reduction in grip strength at both follow-ups (13, 14). Randomized controlled trials by Courneya et al. (15) and Hornsby et al. (16) demonstrated that breast cancer patients receiving adjuvant and neoadjuvant chemotherapy showed significant declines in exercise capacity measured by 5) oxygen uptake during peak exercise (VO_2peak_). In healthy women, VO_2peak_ decreases by 10% every decade, and these studies reported a reduction of a similar magnitude induced by short-term chemotherapy, suggesting that chemotherapy causes a decade’s equivalent of physiologic aging, at least for the effect studied.

The American Society of Clinical Oncology Guideline for Geriatric Oncology recommends a geriatric assessment (GA) for the early identification and treatment of areas of vulnerability for patients at least 65 years old receiving chemotherapy (17). The GA evaluates seven domains: functional and physical status, objective physical performance, comorbid medical conditions, cognition, nutritional status, psychological status, and social support. Each domain is an independent predictor — other than chronologic age — of morbidity and mortality in older cancer patients (17, 18). Moreover, compared with usual care, inclusion of GA in oncology clinic visits for older adults with cancer significantly improves patient-centered and caregiver-centered communication about aging-related concerns (19).

Use of GA has shown positive outcomes in several studies. Li et al. revealed that completing the GA with cancer patients ≥65 years old diagnosed with solid tumors significantly decreased chemotherapy toxicity compared with usual care (50% vs. 60%) (20). Mohile et al. showed significantly reduced chemotherapy toxicity (measured by the number of patients over 3 months with a grade 3 to 5 toxic effect) based on the National Cancer Institute (NCI) Common Terminology Criteria for Adverse Events (Version 4) when GA was used for cancer patients >70 years old with incurable solid tumors or lymphoma (50% vs. 71%) to evaluate age-related domains in these patients and guide management accordingly (21). Soo et al. showed that cancer patients ≥70 years old diagnosed with solid tumors and lymphoma had improved quality of life, 41% fewer hospitalizations, and 39% fewer visits to the emergency department when GA was administered compared with usual care (22). Nipp et al. showed significantly decreased length of hospital stays (8.2 vs. 7.3 days) and decreased intensive care unit admissions (32% vs. 13%) in cancer patients ≥65 years old undergoing surgery for gastrointestinal cancer when the GA was administered versus usual care (23).

These compelling GA findings on reduction of symptomatic toxicities also apply to older cancer survivors who complete curative-intent chemotherapy and support the hypothesis that cancer treatment impacts aging. Indeed, older breast cancer patients treated with chemotherapy were at an elevated risk for post-treatment cognitive decline and factors associated with cognitive aging, such as low cognitive capacity and apolipoprotein status (24). The GA is vital to assess function in patients before cancer treatment to tailor the cancer treatment to their functional status. A recently funded NCI R01 study (CA249457) will examine whether a GA model of care can improve functional and cognitive outcome trajectories in older cancer survivors completing chemotherapy in a large, nationwide cluster-randomized study.

## Mechanisms of accelerated aging in cancer survivors

Multiple cellular mechanisms, including genomic instability, telomere attrition, stem cell exhaustion, epigenetic alterations, and cellular senescence, drive biologic aging (25). Importantly, biologic aging is malleable and is accelerated by stressors such as cancer therapy, which may account for early aging in cancer survivors (26). Indeed, many studies have shown that cancer treatment modalities can induce these aging hallmarks (Table 2), and the contributions of these cellular mechanisms to survivor aging have also been described (Table 3).

### Genomic instability.

Genomic instability, featuring somatic mutations, chromosomal aneuploidies, and copy number variations, increases with physiologic aging as DNA damage accumulates and the capacity of DNA repair mechanisms declines (27). The age-related outcomes associated with genomic instability include cancer, neurologic disease, and osteoarthritis. DNA damage can be measured by immunostaining for γH2AX, which accumulates at the damage site (28).

Cancer treatments introduce genomic instability. DNA damage caused by free radicals generated during chemotherapy increases the risk of leukemogenesis and secondary cancers (29). Alkylating agents are notorious in this regard; they significantly increase the risk of developing leukemia, either alone or in combination with other agents such as epipodophyllotoxins (30, 31). Therapeutic agents such as etoposide and teniposide inhibit DNA repair enzymes (e.g., topoisomerase II), leading to genomic instability. Topoisomerase II inhibitors are also associated with an increased risk of developing second cancers, such as secondary leukemia (32) and second primary leukemia (33). Radiotherapy-induced DNA damage is strongly associated with an increased incidence of cancers. Although advancements in technique and machinery have helped, the likelihood of developing post-irradiation secondary cancer is still much higher than after chemotherapy (34, 35).

### Telomere attrition.

Telomeres are located at the ends of chromosomes and shorten with each replicative cycle until the cell reaches its “Hayflick limit,” after which it undergoes senescence or apoptosis (36). Telomere length decreases with age, making it a marker of aging (37). Excessive telomere attrition is associated with numerous adverse clinical outcomes, including coronary heart disease, hypertension, obesity, metabolic syndrome, cancer, all-cause mortality, and osteoporosis and osteoarthritis (38).

Cancer therapies induce telomere attrition, setting up cancer survivors for accelerated aging. The kinetics of hematopoietic cell transplantation (HCT) causes replicative stress on the stem cell lines of recipients, with consequent stem cell exhaustion and shorter telomeres than those of donors (39). However, telomere shortening after HCT may be temporary, with a return to normal length afterward (40). Chemotherapy also causes telomere shortening by directly affecting telomere length or inhibiting telomerase, the enzyme that maintains telomeres. For example, cisplatin directly inhibited telomerase activity in treatment of primary hepatocellular carcinoma, resulting in a mean decrease in telomere length (41). Drugs such as imetelstat inhibit telomerase in murine studies and various cancer cell lines (42).

### Epigenetic alterations.

Epigenetic modifications, particularly DNA methylation (DNAm) variations of specific CpG dinucleotides, occur with aging and can be measured from blood samples by epigenetic clocks. The first clocks discovered, the Horvath (43) and Hannum (44) epigenetic clocks, predict chronologic age as an outcome measure by generating a weighted average of DNAm age (i.e., an aggregate of CpG DNAm patterns in a given sample) using a linear regression model. Levine’s PhenoAge predicts phenotypic age by replacing chronologic age as an outcome measure with a surrogate marker of biologic age. Therefore, PhenoAge is better predictive of the development of age-related phenotypes such as frailty, cognitive impairment, and chronic diseases, including cancers (45). Lu et al. developed GrimAge, which is highly predictive of phenotypic age and mortality risk (46). Epigenetic age acceleration (EAA) — individuals epigenetically older than their chronologic age — predicts the onset of age-related conditions such as frailty, age-related dementia, impaired cognitive performance, cancers, cardiovascular diseases, and neurodegenerative diseases as well as all-cause mortality (47–49).

Cancer therapies disrupt the epigenome. Topoisomerase II inhibitors, microtubule inhibitors, doxorubicin, cross-linking agents, and methotrexate induce DNA hypermethylation, accelerating aging (50). Furthermore, epigenetic modifiers such as DNA methyltransferase inhibitors (5-aza-2′-deoxycytidine [5-aza]), histone deacetylase inhibitors (panobinostat, vorinostat), histone acetyltransferases (curcumin), and histone methyltransferases (BRD4770) induce senescence in preclinical models (51, 52). For instance, 5-aza induces senescence in osteosarcoma, liver cancer, and lung mesothelioma cells, shown by p16^INK4^, senescence-associated β-galactosidase, and DNA damage response (DDR) signaling (53, 54). Another drug, vorinostat, is FDA-approved for treating cutaneous T cell lymphoma but induces senescence in leukemia, colon, and urothelial cancer cell lines (55–57). While drugs such as azacytidine and vorinostat are FDA-approved as anticancer drugs, clinical evidence regarding their role in senescence and aging is lacking. Ongoing clinical trials evaluate the use of azacytidine in various malignancies (58), and only their completion will reveal its longitudinal effect on the human aging phenotype.

Only a few studies have evaluated EAA in cancer survivors. Significant EAA occurs on Hannum, PhenoAge, and GrimAge clocks in breast cancer patients after radiotherapy or chemoradiation (59). However, evidence on EAA and adverse health outcomes in survivors is currently limited. A recent study reported no EAA — measured by the skin-blood clock — in CCS at the end of chemotherapy; in fact, there was a statistically significant reduction in epigenetic age of 1.1 years (60). However, in adult CCS (average time after cancer diagnosis, 22 years), statistically significant EAA of +5.5 years was detected, but this was independent of DNAm alterations. The GrimAge methylation clock did not show EAA in adult CCS, except in adult CCS who died, in whom GrimAge showed statistically significant EAA of +8.8 years in comparison with non-deceased CCS (60).

Understanding the role of epigenetic alterations in aging-related clinical outcomes has encouraged interventions to help minimize these modifications (61). However, certain lifestyle factors, such as obesity and smoking, can also influence cellular aging (62, 63). In addition, dietary habits also affect cancer and aging through epigenetic alterations linked to the formation and progression of various neoplasms (64, 65). Therefore, it is challenging to do a controlled study on an epigenetic modifier, since lifestyle factors are difficult to control and can become potential confounders.

### Stem cell exhaustion.

Stem cells are depleted with aging (66). Furthermore, stem cell exhaustion results in clonal hematopoiesis, increasing the risk of hematogenous malignancies and all-cause mortality. Cancer therapies such as doxorubicin and daunorubicin can induce stem cell exhaustion. HCT can also cause stem cell exhaustion, likely due to replicative stress during hematopoietic reconstitution, which shortens telomeres, causing stem cell exhaustion (67). A study on primates found that replicative stress after HCT skews hematopoiesis and delays recovery of cell lines, with chronic changes in the BM, increasing future cancer risk (68). In the elderly, this risk is amplified by the replicative stress of HCT compounded by a decline related to physiologic aging (69). In line with these results, the use of younger HCT donors was associated with a significantly increased 5-year survival rate and lower incidence of diseases such as graft-versus-host disease (70). Additionally, a single serial HCT almost doubles cellular age in recipients (71), so younger donors are always preferred to their older siblings.

### Cellular senescence.

**Figure 1:**
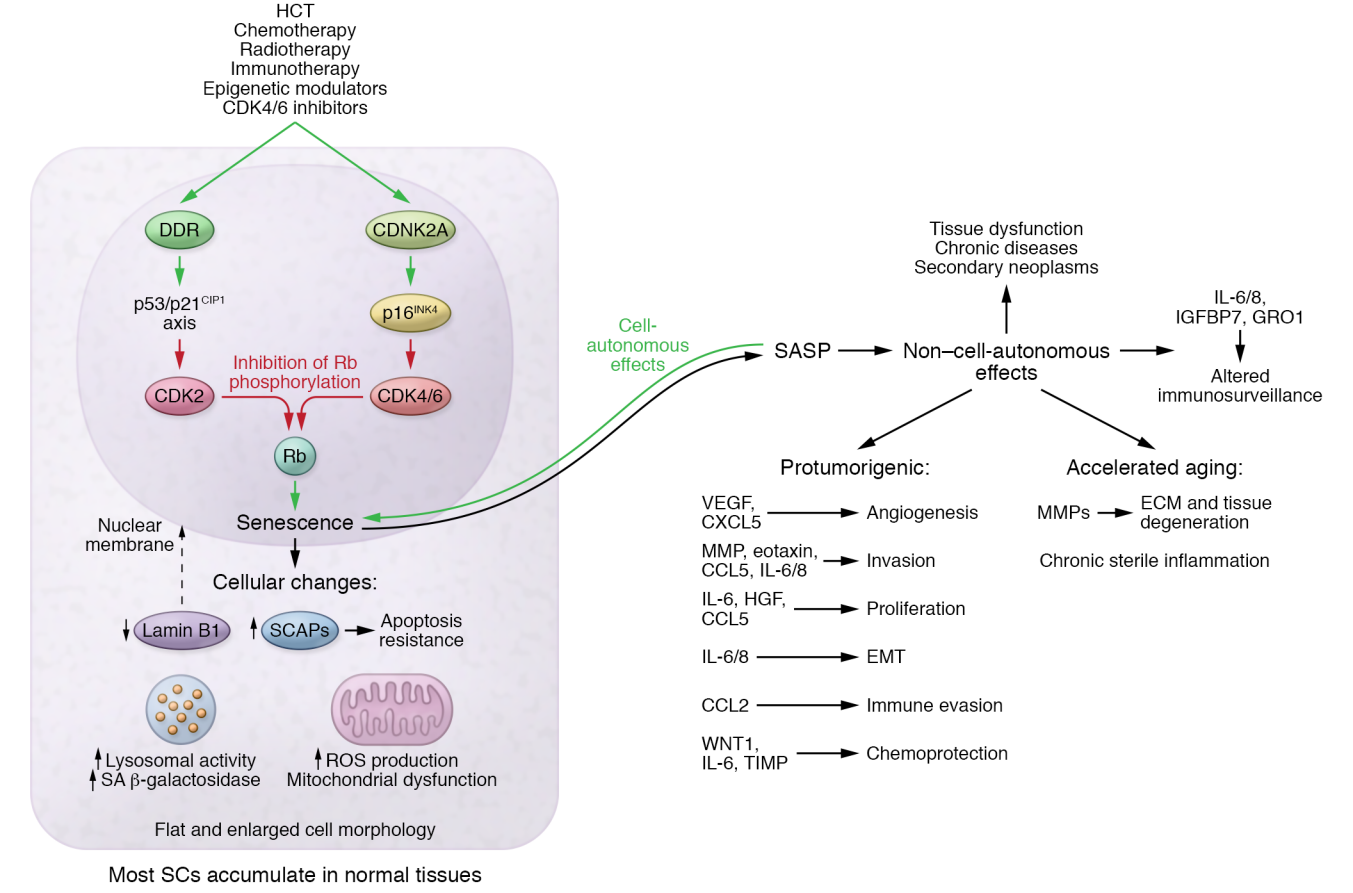
Cancer therapies can induce senescence via two pathways. The replicative senescence pathway is initiated by a DDR that triggers the p53/p21^CIP1^ axis and inhibits CDK2. Alternatively, oncogene-induced senescence is triggered by activation of the *CDNK2A* gene locus encoding p16^INK4^, which inhibits CDK4/6. Both senescence-mediating pathways converge by inhibiting phosphorylation of the Rb protein, which, in turn, causes senescence. Senescent cells release a characteristic secretome termed the SASP, components of which reinforce senescence in an autocrine fashion, termed cell-autonomous effects. Moreover, SASP factors exert non-cell-autonomous effects on neighboring and distant cells. In this regard, they can also mediate ECM degradation, chronic sterile inflammation, and immunosenescence. The resulting tissue dysfunction manifests clinically as accelerated aging phenotypes and a higher burden of chronic diseases, including cancer. Indeed, a higher senescent cell burden may be responsible for these aging phenotypes being observed in higher frequencies in cancer survivors, as compared with healthy controls without a history of cancer.

Cellular senescence is a cell fate of growth arrest described initially by Hayflick and Moorhead in fibroblasts (72, 73). Senescent cells (SCs) feature many alterations at the cellular level, including proliferative arrest, resistance to apoptosis via upregulation of senescence-associated antiapoptotic pathways (SCAPs), chromatin alterations, and metabolic and synthetic changes (Figure 1) (74). The senescence-associated secretory phenotype (SASP), a characteristic transcriptomic signature expressed by many SCs of proinflammatory cytokines, chemokines, growth factors, and proteases, allows SCs to influence the tissue microenvironment in autocrine (cell-autonomous effects) and paracrine (non-cell-autonomous) manners (75, 76).

Senescence has no specific marker. Senescence-associated β-galactosidase (SA-β-gal), a lysosomal enzyme that accumulates in SCs, is commonly used to define a senescent state (77). Mediators of senescence — p16^INK4^, p21^CIP1^, p53, and p14^ARF^ — can indicate growth arrest. Downregulation of the nuclear lamina protein lamin B1 is also a common feature of SCs (78). The DDR markers γH2AX and 53BP1 can be immunostained to identify SCs (79), but γH2AX foci within telomeric DNA, termed telomere-associated foci (TAFs), are more specific for SCs. Studies also monitor SASP components such as IL-6 and IL-8. Since we still lack a single sensitive and specific biomarker, studies use combinations of the above biomarkers to monitor SC burden.

Several anticancer therapies induce senescence. Therapy-induced senescence (TIS) is a well-established response to chemotherapy and radiotherapy. Chemotherapeutic agents damage DNA to cause cell death by apoptosis, but sub-cytotoxic doses activate a DDR that drives cells into senescence. Chemotherapeutic agents also shorten telomeres, which may induce replicative senescence. In this instance, doxorubicin induces senescence in fibroblasts, vascular smooth muscle cells, and numerous cancer cell lines, demonstrated by elevated SA-β-gal and p16^INK4^ upregulation or p21/p53 signaling (80). Furthermore, treatment with cyclophosphamide, adriamycin, and 5-fluorouracil increases SA-β-gal expression in 41% of human breast cancer tissues (81).

Radiotherapy causes cell death via ROS-mediated DNA damage, which may drive the cell into apoptosis or senescence if irreparable. Ionizing radiation (IR) is commonly used to induce senescence in murine models. IR-treated cells, including breast cancer, colon cancer, neuroblastoma, and fibrosarcoma cell lines, exhibit numerous senescence markers, including SA-β-gal, p16^INK4^, p21, p53, and SASP expression (80). In CCS who received cranial radiation, biopsies from the scalp show significantly higher expression of senescence markers than biopsies taken from other body areas not irradiated, such as the buttocks (82). Exposure to cancer therapy elevates senescence biomarkers in cancer survivors by magnitudes comparable to several years of chronologic aging (Table 3).

## Cellular senescence: the Achilles’ heel of cancer survivors

Cancer survivors are at a significantly higher risk of age-related diseases than non-cancer controls, comparable to incident rates in the elderly population. Cellular senescence is a biologic aging hallmark and plays a causative role in numerous age-related diseases, many of which affect cancer survivors. Furthermore, many cancer therapies induce senescence, suggesting that TIS may be responsible for cancer survivors’ various side effects.

The seminal study of Demaria et al. showed that treating fibroblasts with doxorubicin induces senescence, as indicated by higher SA-β-gal, p16^INK4^, p21^CIP1^, and DDR expression, elevated IL-1α, IL-6, MMP-3/9, CXCL1, CXCL10, and CCL20, and reduced lamin B1 (83). Notably, doxorubicin induces senescence systemically and not only in tumor cells, as indicated by an increase in whole-body bioluminescence after doxorubicin treatment. In addition, doxorubicin significantly impairs hematopoietic stem cell function by reducing the number of colony-forming units, an effect rescued by ganciclovir-mediated (GCV-mediated) clearance of SCs (83). Furthermore, cardiomyopathy, a well-known side effect of doxorubicin, was almost entirely prevented by GCV treatment. Treating mouse breast cancer models with doxorubicin arrests tumor growth, with later cancer relapse, but combining doxorubicin with GCV significantly improves the survival of mice, reduces the incidence of metastasis, and reduces the number of metastatic foci in mice that developed metastasis. Lastly, the nocturnal running time of mice was significantly impaired after doxorubicin treatment, and GCV treatment almost entirely rescued this effect (83).

Eliminating SCs alleviates many acute effects (elevated inflammatory markers and cardiotoxicity) and chronic effects (fatigue, cancer relapse, metastasis) of doxorubicin, suggesting TIS-dependent pathogenesis of cancer therapy–related adverse effects in survivors, at least those treated with doxorubicin. Mechanistic insights into how SCs may contribute to these pathologies are discussed below.

### Senescence and aging.

SCs accumulate in aging tissues, and senescence biomarkers increase with age at sites of age-related pathologies, including atherosclerosis, osteoarthritis, idiopathic pulmonary fibrosis (IPF), and age-related metabolic dysfunction. Transplantation of a relatively low dose of SCs such that the ratio of SCs to non-SCs is 1:10,000 is sufficient to induce frailty, accelerated aging, and early death. The association between SC accumulation and decreased lifespan began in 2004, when a study in mice reported that caloric restriction increased lifespan by delaying accumulation of p16^INK4^ and SA-β-gal (84). In transgenic INK-ATTAC mice, in which SCs can be selectively ablated, SC depletion alleviated numerous age-related disorders, including sarcopenia, cataracts, and cachexia, and increased lifespan (85, 86).

Mechanistically, a two-part model explains the association between senescence and aging. Firstly, senescence in stem cells with aging arrests proliferation, decreasing tissue regeneration. In INK-ATTAC mice, the loss of self-renewal capacity of muscle satellite cells and fat progenitor cells due to cellular senescence drives loss of sarcopenia and loss of adipose tissue mass, respectively, and clearance of SCs alleviates these phenotypes (86). Secondly, SCs influence their tissue microenvironment in a paracrine fashion via the SASP. For instance, matrix metalloproteinases (MMPs) drive ECM damage and tissue degeneration, such as loss of elasticity in skin and lung. Furthermore, IL-1α, IL-6, IL-8, CCL2, and CXCL12 contribute to fatigue, cardiovascular morbidity, and appetite loss. In addition, IL-1β, TNF-α, and IL-6 mediate peripheral IGF-1 resistance, resulting in sarcopenia and reduced cardiac function. Lastly, SASP factors cause sterile inflammation, resulting in tissue fibrosis and degeneration (87).

SCs are also present at sites of age-related pathologies, including atherosclerosis, diabetes, glaucoma, osteoarthritis, and IPF, among others. But whether SCs causally underlie these pathologies or are simply the consequence of them remains an area of active investigation. SCs are intrinsically resistant to apoptosis, making their elimination challenging. A seminal study hypothesized that SCs evade cell death by upregulating SCAP networks, meaning that inhibition of SCAPs would selectively eliminate SCs (88). Transcriptomic analysis has revealed upregulation of SCAPs in senescent preadipocytes compared with non-senescent cells, and using siRNAs to inhibit SCAPs has selectively eliminated SCs (88). In vitro, a combination of dasatinib and quercetin (D+Q) eliminated SCs. Dasatinib is a pan–tyrosine kinase inhibitor used in cancer treatment, whereas quercetin inhibits PI3K. The use of D+Q in a murine model selectively eliminated SCs, as indicated by reduced p16^INK4^ mRNA expression and SA-β-gal–positive cells (88). Inducing senescence in one leg of wild-type mice by radiation and then treating with D+Q reduced p16^INK4^ mRNA expression in muscle and SA-β-gal–positive fat cells in the leg, with resultant improved exercise capacity (88). Additionally, treating mice with a D+Q regimen significantly improved cardiac function and carotid vascular reactivity and delayed age-related pathologies such as osteoporosis and intervertebral disc degeneration (88). Recent preclinical studies affirm that eliminating TIS cells alleviates the pathology of diabetes, obesity, cardiac dysfunction, frailty, Alzheimer’s and Parkinson’s diseases, osteoporosis, osteoarthritis, and IPF, among others (89–98). The mechanisms, at least those elucidated in murine models, by which cellular senescence contributes to these pathologies are reviewed elsewhere (87). Nevertheless, observing that SC elimination alleviates numerous age-related pathologies implicates a senescence-dependent pathogenesis to these phenotypes, and similar mechanisms could underlie early aging phenotypes in cancer survivors.

### Protumorigenic cell-autonomous effects of senescence.

Since SCs are intrinsically resistant to apoptosis, TIS cancer cells may persist, leading to tumor dormancy and higher chances of cancer relapse in the future (99, 100). Studies have shown that prolonged culture of TIS cells eventually results in senescence escape and cell cycle reentry with higher expression of stemness markers (101). In this regard, doxorubicin-induced senescence in mouse lymphoma models temporarily arrested tumor growth but upregulated stemness markers, including *WNT* signaling, as manifested by some cells resuming proliferating and showing increased aggressiveness (102).

CDK1 (encoded by *CDC2*) mediates cell cycle reentry. In non–small cell lung cancer cells, chemotherapy-induced senescence temporarily arrested growth, but cells soon resumed proliferation by activating *CDC2*, while CDK1 inhibitors or knockout of *CDC2* prevented escape from TIS (103). Genomic instability is another important mediator of cell cycle reentry: doxorubicin-treated colon cancer cells that escaped senescence exhibit aneuploidy, whereas euploid TIS cancer cells do not escape senescence (104). Lastly, p53-dependent senescence and apoptosis resistance significantly contribute to treatment refractoriness and recurrence after completion of therapy. Wild-type p53 mammary tumors display a poorer response to doxorubicin than those harboring mutated p53 (105). Whereas the latter continue proliferation, leading to abnormal mitosis and cell death, wild-type p53–bearing tumors undergo senescence in response to chemotherapy, resulting in the production of the SASP factors eotaxin and CCL5, which promote tumor relapse (105).

### Protumorigenic non-cell-autonomous effects of senescence.

SCs possess the ability to influence their microenvironments via their SASP to drive various aspects of tumorigenesis. These processes derive from observations in preclinical mouse models, in which coculturing preneoplastic or overtly cancerous cells with senescent fibroblasts enhances proliferation, angiogenesis, invasion, and epithelial-mesenchymal transition (EMT).

Culturing mouse premalignant and malignant epithelial cells with senescent human fibroblasts accelerates tumor growth (106). SASP components IL-6 and IL-8 activate *STAT3*, a critical oncogene, mediating numerous tumorigenic effects of the SASP. STAT3 induces the expression of c-myc, c-fos, cyclin D1, and mTORC1 to drive proliferation (107). VEGF, another SASP component, causes angiogenesis, and coinjection of senescent fibroblasts with cancerous epithelial cells promotes angiogenesis (108). Another crucial aspect of carcinogenesis is local invasion, predominantly driven by MMPs (108). STAT3 also drives the transcription of MMPs. Indeed, breast cancer cells are more invasive when cocultured with senescent fibroblasts (109). EMT is an essential hallmark of carcinogenesis characterized by tumor cells acquiring migratory capabilities. SASP components IL-6 and IL-8 promote EMT via STAT3, which decreases the expression of the surface adhesion molecules E-cadherin and β-catenin (109–111). IL-6 has also been shown to promote osteolytic metastasis of breast cancer by stimulating osteoclastogenesis, and neutralization of IL-6 was sufficient to prevent this occurrence (112).

These processes are exemplified by a report that the use of doxorubicin to induce senescence in a murine breast cancer model results in release of the SASP factors eotaxin, CXCL5, and CCL5, which promote tumorigenesis manifested as cancer relapse (105). Eotaxin promotes invasion through MMP3 upregulation, CXCL5 activates VEGF to stimulate angiogenesis and AKT/GSK3β/β-catenin signaling to stimulate EMT, and CCL5 promotes proliferation by upregulating c-myc and cyclin D1 (105). Accordingly, eliminating doxorubicin-induced SCs reduces tumor growth and cancer relapse (83). Another study demonstrated that a well-established two-step carcinogenesis protocol of DMBA and TPA administration promotes skin carcinogenesis — specifically the progression of benign papillomas to invasive squamous cell carcinoma (SCC) of the skin — in a senescence-dependent manner via p38/MAPK/ERK signaling. Furthermore, eliminating SCs reduces p38/MAPK/ERK signaling and prevents the progression of benign papillomas to SCC, showing that senescence induction plays a role in tumor promotion (113).

Immune evasion is another critical effect of SASP factors. Non-tumor cells affected by TIS secrete a SASP comprising WNT16B, IL-6, and TIMP-1, protecting cancers from chemotherapy (114). In addition, CCL2 in hepatocellular carcinoma (HCC) models attracts CCR2^+^ myeloid cells that stimulate SC clearance via immunosurveillance but can promote the growth of already established HCC cells by inhibiting NK cell–mediated clearance of cancer cells (115). Therefore, senescence may create a local immunosuppressive environment favoring the persistence of tumor cells. Indeed, coinjecting senescent fibroblasts with skin carcinoma cells into mice increases tumor growth via infiltration of immunosuppressive myeloid cells, while this effect is absent in immunocompromised mice (116).

## Senotherapeutics

Two strategies exist for targeting senescence: eliminating SCs through senolytics that inhibit SCAPs, and alleviating phenotypes of SCs by senomorphics, which inhibit the SASP. These drugs are primarily adjuncts to chemotherapy, intended to eliminate therapy-induced SCs (senolytics) or mitigate SC effects (senomorphics) — a strategy termed “one-two punch” cancer therapy (117). Notably, senescence is physiologically crucial in wound healing (118, 119), embryogenesis (120, 121), and initiation of labor (122). Therefore, senotherapies should ideally combat the pathologic effects of SC accumulation while sparing these physiologically beneficial aspects. Lastly, the establishment of senescence takes several weeks, which underlies the “hit-and-run” principle of senolytic therapy: senolytic drugs administered intermittently over extended time intervals are just as effective as continuous administration.

### Clinical data on the efficacy of senolytics.

The first senolytics selected using bioinformatics approaches were dasatinib, an approved chemotherapy drug, and quercetin, a naturally occurring flavonoid (88). Fisetin is another naturally occurring flavonoid closely related to quercetin but with a shorter half-life. In vitro studies evaluating fisetin as a senolytic revealed antiproliferative and proapoptotic effects (123, 124). The short half-life of these drugs is in line with the hit-and-run principle. Indeed, intermittent administration of dasatinib and quercetin (D+Q) is as effective as continuous dosing, suggesting a direct cytotoxic effect rather than receptor occupancy or enzyme inhibition (125). Lastly, navitoclax inhibits BCL-2, promoting apoptosis of SCs (126). Unlike the other senolytics, navitoclax targets a specific SCAP, whereas dasatinib, quercetin, and fisetin target SCs and not a pathway; i.e., these drugs were not developed using the classic one target, one drug, one disease model. This distinction accounts for navitoclax’s unfavorable adverse effect profile, as it targets non-SCs expressing BCL-2, particularly platelets, causing thrombocytopenia (127). However, conjugating navitoclax with SA-β-gal increases specificity for SCs and reduces platelet toxicity (128). Conventional high-throughput library screens identify second-generation senolytics, and many now exist (129).

Since senolytic drugs constitute a novel therapeutic modality, the initial clinical trials using these drugs were restricted to severe treatment-refractory conditions (89). In this context, the first study reported significantly improved physical health measures in IPF patients receiving intermittent D+Q, as measured by 6-minute walk distance, 4-meter gait speed, and chair-stand time (130). Another phase I trial administered D+Q to diabetic kidney disease patients and sampled adipose tissue before treatment and 11 days after treatment to evaluate SC burden. Indeed, p16^INK4^, p21^CIP1^, and SA-β-gal expression decreased in post-treatment adipocytes. In addition, a panel of circulating SASP factors showed a decrease in levels of IL-1α, IL-6, MMP-9, and MMP-12 (131).

Numerous clinical trials are currently under way evaluating senolytics in cancer survivors. A study assessing the efficacy of D+Q therapy in decreasing SC burden in HCT survivors, manifested in lower levels of senescence biomarkers, is currently ongoing at the Mayo Clinic (ClinicalTrials.gov NCT02652052). Two clinical trials, AFFIRM (NCT03430037) and AFFIRM-LITE (NCT03675724), are investigating fisetin for alleviation of frailty and associated disorders in older women and older adults, respectively.

## Discussion

Focusing on cellular senescence over other mechanisms assumes that senescence drives accelerated aging processes in cancer survivors while conferring a relatively limited role to other biologic aging hallmarks. This, however, has not been proven; but since transformative preclinical advancements in alleviating age-related health conditions have been achieved by elimination of SCs, we feel it appropriate to focus our Review on cellular senescence and advocate that considering cellular senescence as the driver of early aging in survivors could have great benefits in advancing the implementation of potential cutting-edge interventions to mitigate premature aging.

However, phenotypic differences between physiologic aging and early aging in survivors are not yet apparent, nor are the distinctions in the molecular mechanisms driving them; differences between physiologic aging and survivor aging need to be anticipated. Furthermore, physiologic aging is characterized by disparities in organ-specific aging, reflecting inherent variation in tissue susceptibility to aging (132). In survivors, chemotherapy and radiotherapy may also differentially affect organ systems, leading to organ-specific aging phenotypes; this may also be dependent on the specific treatment survivors receive. Another problem is rationalizing the limitation of trials to specific biomarkers over others. Since it is impossible to be all-inclusive, preclinical research must provide the groundwork for associating specific aging biomarkers with organ-specific outcomes. Regarding senescence, the relative contributions of different senescence-inducing pathways to specific age-related diseases remain undetermined. A very recent study showed that radiation-induced osteoporosis in mice is mainly driven by p21^CIP1^ SCs rather than SCs expressing p16^INK4^ and that eliminating p21^+^, but not p16^+^, cells ameliorates this pathology (133). These comparative mouse models should be used by future research to associate specific senescence pathways with organ-specific aging outcomes, which will help rationalize the use of specific biomarkers over others.

SC heterogeneity — whereby SCs differ in their phenotype based on cell type, tissue of origin, nature of the senescence-inducing stimulus, and time elapsed since the insult — needs to be better characterized. This heterogeneity manifests in varying SASP compositions and in the use of different SCAPs by SCs to evade death, affecting therapy response (117). SC heterogeneity can limit the indication of certain senotherapies to specific cancer types, as well as limit the generalized efficacy of senolytics in clinical trials, since TIS cells in other cancer types may exhibit different properties (134, 135). Heterogeneity in the expression of SC markers also complicates the detection and monitoring of SC activity. A recent study showed that even though the pharmacologic CDK4/6 inhibitor abemaciclib induces p53-dependent senescence, these TIS cells elaborate a SASP lacking proinflammatory factors and thereby lack the various protumorigenic effects of the SASP, while still retaining its antiproliferative and immunosurveillance impact (136). This is in line with the notion that cancer patients tolerate CDK4/6 inhibitors better than standard chemotherapies.

The lack of a specific biomarker of senescence hinders effective monitoring of SC burden, and limits accurate characterization of SC heterogeneity. Moreover, tissues such as muscle cells do not appear to express p16^INK4^ or p21^CIP1^ (137). The prognostic significance of monitoring SC burden in cancer survivors is not fully understood, and long-term clinical studies assessing patients currently enrolled in trials are required. Furthermore, biomarkers of senolysis are needed to better evaluate the effects of senolytics. A recent study demonstrated that oxylipin 15-deoxy-Δ12,14-prostaglandin J2, a particular oxylipin (138), accumulated inside SCs and was released upon their elimination, suggesting its utilization as a senolysis biomarker (138).

Although epigenetic age and senescent biomarkers constitute potential endpoints for clinical trials evaluating the efficacy of senotherapies, lifestyle and environmental differences other than chronologic age and cancer therapy also influence epigenetic age. A recent systematic review and meta-analysis on social, environmental, and biologic accelerators of epigenetic aging revealed male sex, alcohol consumption, low education level, low socioeconomic status, high BMI, diabetes, and smoking to accelerate epigenetic aging on Horvath, Hannum, PhenoAge, and GrimAge aging clocks (139). How this multitude of factors can confound the results of studies using epigenetic aging as an endpoint of aging in cancer survivors remains undetermined. Clinical trials using epigenetic clocks to evaluate the effect of senolytics must control for these variables.

The senescence-inducing capabilities of immunotherapies remain unelucidated in cancer but are theoretically likely due to their apoptosis-inducing mechanism of action. For example, rituximab, an anti-CD20 monoclonal antibody, induces senescence in B cell lymphoma, manifested as increased SA-β-gal expression, p21/p53 signaling, and elaboration of a SASP (80, 140). Therefore, future research should focus on whether and how immunotherapies induce senescence, and accelerate aging in various specific cancer types, as the number of cancer survivors who receive immunotherapeutic regimens will only increase in the following years.

The relevance of accelerated cellular senescence also remains unexplored in cancer patients treated surgically. Senescence plays a crucial role in physiologic wound healing. PDGF-AA, a SASP component, induces collagen production and wound contraction by activating myofibroblasts. MMPs are essential to wound remodeling. Senescence induction may also prevent fibrosis. Accordingly, SC depletion in mouse models impairs wound healing (118). However, while transient senescence is beneficial, chronic senescence dysregulates wound healing (119). A two-pronged approach needs to be adopted when associating senescence and outcomes in surgically treated cancer patients, i.e., the effects of both a high SC burden and senotherapies.

Undoubtedly, there is a concerted effort from the scientific community to address the phenotypes, mechanisms, biomarkers, and interventions of early aging in cancer survivors. However, there has been much hype concerning therapeutics and misuse of the so-called anti-aging agents without conclusive evidence of safety and efficacy. Knowledge about cellular senescence has exponentially increased in recent years on the basis of preclinical studies, but only the outcomes of well-designed, robust clinical studies can prove whether senotherapies will be beneficial in decreasing morbidity, increasing longevity, and improving quality of life in survivors. Thus, the scientific community must go through the rigorous process of translating bench work into clinical trials with a well-defined outcome. Only after completion of randomized trials, if senolytics and other anti-aging drugs show excellent short- and long-term safety and efficacy, should these drugs be used in the clinic.

## Author contributions

Under the supervision of SKH, SS and AS wrote the first draft; all authors approved the final version of the manuscript.

## Figures and Tables

**Table 3:**
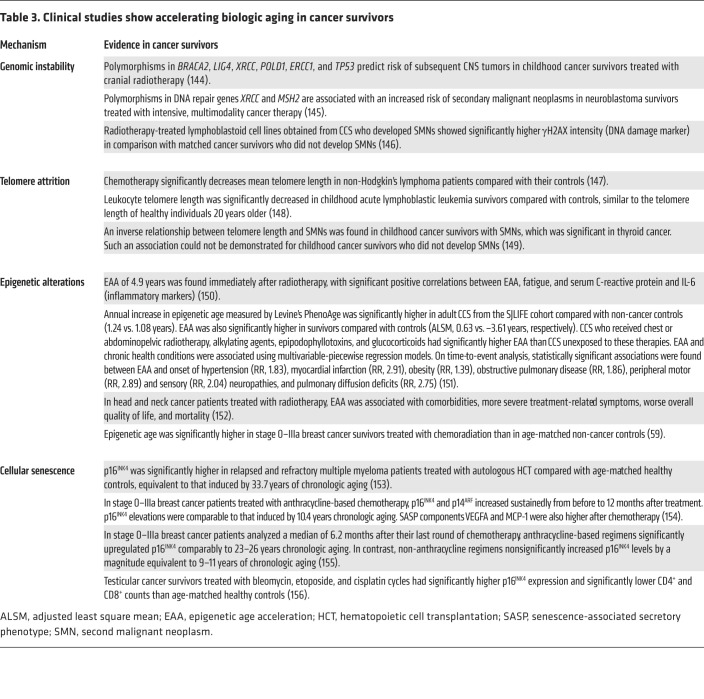
Clinical studies show accelerating biologic aging in cancer survivors

**Table 2:**
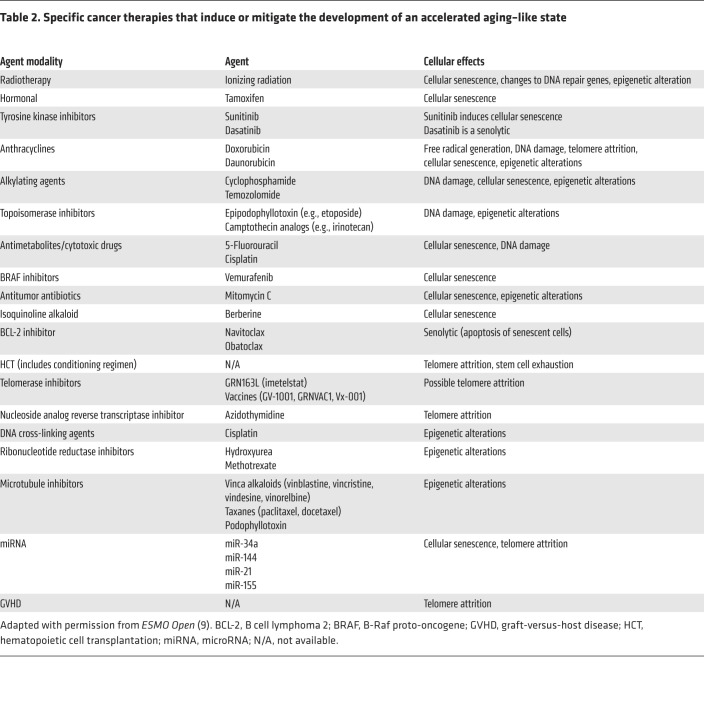
Specific cancer therapies that induce or mitigate the development of an accelerated aging–like state

**Table 1:**
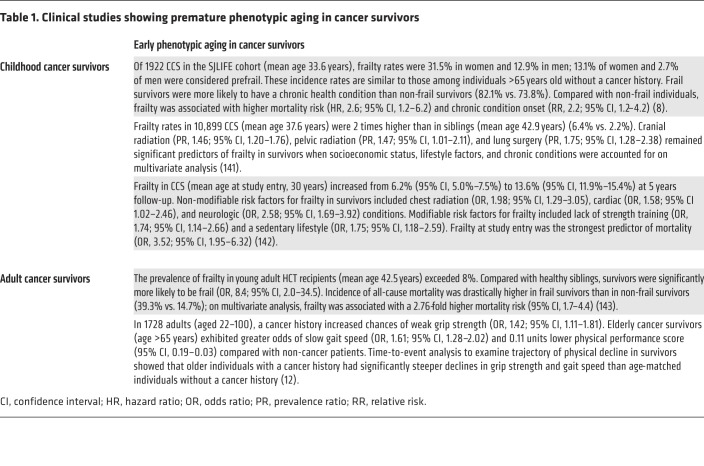
Clinical studies showing premature phenotypic aging in cancer survivors

## Version 1 07/01/2022

Electronic publication
